# Supplementary material for: Detailed molecular and epigenetic characterization of the pig IPEC-J2 and chicken SL-29 cell lines
Source: iScience. 2023 Feb 20;26(3):106252. doi: 10.1016/j.isci.2023.106252 (PMC10018572; doi:10.1016/j.isci.2023.106252)
Supplement: Data S2. Complete homer output for identified motifs in Chicken SL-29, related to Tables 5 and 6 — Homer motif analysis results for histone modifications H3K4me1, H3K4me3, H3K27ac, enhancers, and ATAC-seq of chicken SL-29 cell line. Parameters for possible false positives is as mentioned earlier for S5. [file mmc3.zip › Data_S2/S6/Chicken_SL_29/peak_files_enhancers/homerResults/motif7.similar.html]

motif7

## Information for motif7

G
C
T
A
C
G
T
A
A
G
T
C
G
T
A
C
C
T
A
G
A
T
G
C
G
T
C
A
C
G
A
T
  
Reverse Opposite:  

G
C
T
A
C
A
G
T
T
A
C
G
A
G
T
C
A
C
T
G
T
C
A
G
G
C
A
T
C
G
A
T
  

|  |  |
| --- | --- |
| p-value: | 1e-27 |
| log p-value: | -6.275e+01 |
| Information Content per bp: | 1.693 |
| Number of Target Sequences with motif | 4506.0 |
| Percentage of Target Sequences with motif | 24.34% |
| Number of Background Sequences with motif | 6625.3 |
| Percentage of Background Sequences with motif | 21.01% |
| Average Position of motif in Targets | 150.3 +/- 85.6bp |
| Average Position of motif in Background | 150.2 +/- 87.1bp |
| Strand Bias (log2 ratio + to - strand density) | 0.0 |
| Multiplicity (# of sites on avg that occur together) | 1.13 |
| Motif File: | file (matrix) reverse opposite |

### Similar de novo motifs found

|  |  |  |  |  |  |  |  |
| --- | --- | --- | --- | --- | --- | --- | --- |
| Rank | Match Score | Redundant Motif | P-value | log P-value | % of Targets | % of Background | Motif file |
| 1 | 0.639 | C A G T G A T C G A T C C T A G A T G C G A C T A G C T A T G C G T A C A T C G A T C G A G C T A G C T | 1e-17 | -39.748378 | 0.09% | 0.01% | motif file (matrix) |
